# Implementation of continuous-wave Doppler ultrasound to detect the high-risk foetus in the low-risk mother: lessons from South Africa

**DOI:** 10.1186/s12884-023-05721-3

**Published:** 2023-05-27

**Authors:** Tsakane M.A.G. Hlongwane, Robert C. Pattinson, Anne-Marie Bergh

**Affiliations:** 1 Maternal and Infant Health Care Strategies Research Unit, South African Medical Research Council, University of Pretoria, Private Bag X323 Gezina, Pretoria, 0031 South Africa; 2 Research Centre for Maternal, Fetal, Newborn & Child Health Care Strategies, University of Pretoria, Private Bag X323 Gezina, Pretoria, 0031 South Africa; 3 Department of Obstetrics and Gynaecology, University of Pretoria, Private Bag X323 Gezina, Pretoria, 0031 South Africa

**Keywords:** Continuous-wave Doppler, Antenatal care, Placental insufficiency, Stillbirths, Stages of change, Implementation of screening

## Abstract

**Introduction:**

Detecting the risk of stillbirth during pregnancy remains a challenge. Continuous-wave Doppler ultrasound (CWDU) can be used to screen for placental insufficiency, which is a major cause of stillbirths in low-risk pregnant women. This paper describes the adaptation and implementation of screening with CWDU and shares critical lessons for further rollout. Screening of 7088 low-risk pregnant women with Umbiflow™ (a CWDU device) was conducted in 19 antenatal care clinics at nine study sites in South Africa. Each site comprised a catchment area with a regional referral hospital and primary healthcare antenatal clinics. Women with suspected placental insufficiency as detected by CWDU were referred for follow-up at the hospital. A 35–43% reduction in stillbirths was recorded.

**Methods:**

The authors followed an iterative reflection process using the field and meeting notes to arrive at an interpretation of the important lessons for future implementation of new devices in resource-constrained settings.

**Results:**

Key features of the implementation of CWDU screening in pregnancy combined with high-risk follow-up are described according to a six-stage change framework: create awareness; commit to implement; prepare to implement; implement; integrate into routine practice; and sustain practice. Differences and similarities in implementation between the different study sites are explored. Important lessons include stakeholder involvement and communication and identifying what would be needed to integrate screening with CWDU into routine antenatal care. A flexible implementation model with four components is proposed for the further rollout of CWDU screening.

**Conclusions:**

This study demonstrated that the integration of CWDU screening into routine antenatal care, combined with standard treatment protocols at a higher-level referral hospital, can be achieved with the necessary resources and available maternal and neonatal facilities. Lessons from this study could contribute to future scale-up efforts and help to inform decisions on improving antenatal care and pregnancy outcomes in low- and middle-income countries.

**Supplementary Information:**

The online version contains supplementary material available at 10.1186/s12884-023-05721-3.

## Introduction

The vision of the World Health Organization (WHO) is that ‘every pregnant woman and newborn infant receives good quality care throughout pregnancy, childbirth and the postnatal period’ [1]. ‘Quality’ refers to a health system’s provision of care that enables a positive pregnancy experience. Early antenatal detection of the foetus at risk of stillbirth or being small for gestational age in low-risk pregnant women remains a challenge, as these foetuses are not detected by conventional imaging ultrasound [2]. Studies in South Africa have shown that the majority of stillbirths occur in the antenatal period, with a peak in stillbirths between 32 and 38 weeks’ gestation. There are approximately two stillbirths for every neonatal death [3–5]. A further challenge is how to reduce the number of unexplained intrauterine deaths in resource-limited settings because of the subjectivity of the current available antenatal foetal growth-monitoring tools [2, 6, 7].

In 2017 the Basic Antenatal Care (BANC) Plus programme was launched as the South African response [8] to the WHO recommendations for a positive pregnancy experience [1]. BANC Plus includes a minimum of eight antenatal contacts, with more contacts concentrated in the third trimester. Another potential intervention during the third trimester to further improve the quality of antenatal care (ANC) and reduce perinatal mortality is the routine use of a single Doppler ultrasound assessment of the foetal blood vessels to identify placental insufficiency [2, 9, 10]. The WHO guideline development group indicated the need for a more rigorous evaluation of this screening procedure, particularly in low- and middle-income country (LMIC) settings [1]. The recommendation that future trials should be designed to address modest changes in perinatal outcomes, and should focus on potentially preventable deaths, opened a window for research in this field in South Africa with a view to combining it with the rollout of BANC Plus [7].

Studies reporting on results of implementing continuous-wave Doppler ultrasound (CWDU) at various sites throughout South Africa and four other LMICs have shown a significant reduction in stillbirths when the screening was combined with appropriate follow-up of foetuses identified as being at risk [11–14]. In the biggest study (Umbi9), a CWDU device called Umbiflow™ was implemented for screening at nine study sites. This paper describes the adaptation and implementation of CWDU screening, and shares critical lessons, particularly with regard to healthcare provider training, on the use of CWDU and the preparation for and rollout of the screening.

## Methods

The Umbiflow™ device was developed by the South African Medical Research Council (SAMRC) and the Council for Scientific and Industrial Research (CSIR) and was first tested in the Western Cape Province [15–17]. The device has a digital interface with a visual and sound representation for recognition of the classic umbilical Doppler wave sound. Each pregnant woman’s unique waveform is recorded in real time and saved on a laptop and a printout can be made for her maternity case record. The software interprets recorded Doppler waveforms as low, medium or high risk and standardized indices charts use values according to local and international standards [18].

The part of the Umbi9 trial reported on here is a descriptive study of the processes followed during the introduction and implementation of CWDU screening. Implementation across the nine study sites occurred in a step-wedge fashion in order to allow for stakeholder discussions and study-site planning, adequate training and monitoring per study site, adjustment to local circumstances and local study teams’ familiarization with the equipment and study process [13, 14].

**Fig. 1:**
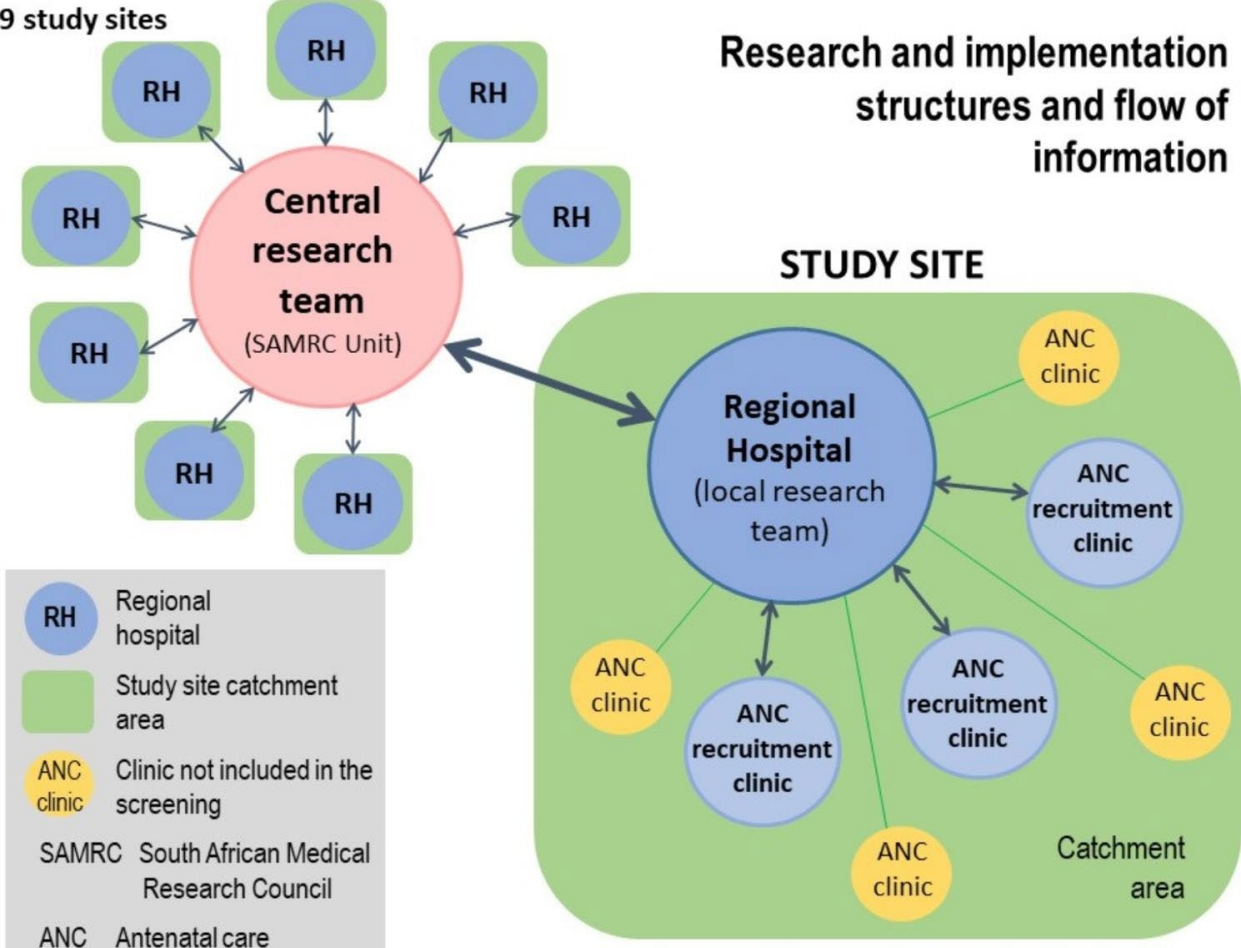
Overview of implementation design

The *research setting* was nine study sites in eight provinces that complied with the inclusion criteria set out in Table 1. Each study site comprised a regional referral hospital and its catchment area. Primary healthcare (PHC) antenatal clinics in the catchment area were purposively selected for CWDU screening according to the following criteria: infrastructure; established referral routes; effective communication; number of pregnant women seen daily; delivery statistics; familiarity with the Perinatal Problem Identification Programme (PPIP) (a clinical audit programme that ensures capture of all perinatal deaths); and availability of transport to connect the different facilities. A total of 19 ANC clinics were included, ranging between one and three clinics per study site. Table S1 in the supplementary file provides more information on the context of each study site and Tables S2 and S3 summarize the characteristics of the clinics and hospitals, respectively, per site. Figure 1 gives an overview of the structure of each study site and the communication and information flow between the central research team and the local research team at the regional hospital.

**Table 1:** Inclusion criteria for study sites

Area	Criteria
**Catchment area**	• Sufficient throughput of pregnant women (number of deliveries and high burden of perinatal mortality) • Baseline data for perinatal and maternal morbidity and mortality and causes of death • Maternity birth register that records pregnancy outcomes • Familiarity with collecting data and using PPIP
**Clinics in catchment area**	• PHC clinics with healthcare professionals offering ANC • Antenatal clinics with a working environment and clinic floor plan that make CWDU examinations feasible
**Referral**	• Established system of referral to the next level of care for women identified as high risk or as having an abnormal RI
**Next level of care** **(referral hospital)**	• Nursing and medical professionals with sufficient knowledge and experience of obstetrics and neonatal care • Pulsed-wave Doppler ultrasound and familiarity with antenatal care for high-risk women • Referral hospital should have - the ability and equipment to take special care of small newborns weighing ≥ 1000 g - facilities to perform caesarean sections - a blood bank and laboratory

ANC, antenatal care; CWDU, continuous-wave Doppler ultrasound; PHC, primary healthcare; RI, resistance index; PPIP, Perinatal Problem Identification Programme

For each study site, a local study nurse and a data clerk were employed. Six study nurses were advanced midwives. The central research team monitored the implementation of CWDU screening of each site weekly by telephone or team-viewer discussion (15–30 min) to get an update on data collection, recruitments and referrals, to review the process and decide on changes, and to facilitate support needs (including stationery and equipment needs). The technical team, through the CSIR, dealt with Umbiflow™ device and digital interface issues, including initial troubleshooting such as testing software settings and adjusting the probe sound.

*Data collection* for describing the process of implementation included the making of field notes by the principal investigator who supervised implementation and notes of meetings and informal discussions with implementers and other stakeholders.

*Data analysis* took the form of iterative discussions between the three authors to arrive at an interpretation of the implementation process that could shed more light on important lessons for future implementation and rollout of CWDU screening.

### Conceptual framework for interpreting the planning and implementation of CWDU screening

The implementation of CWDU screening was triggered by the challenge of finding solutions that would decrease the number of unexplained stillbirths in the maternal population. A stages-of-change model previously used for the planning, interpretation and assessment of other maternal and newborn health initiatives provided guidance on planning the implementation of the Umbi9 project as a whole and implementation at individual study sites. The model has three phases, each with two stages: pre-implementation (create awareness and commit to implement), implementation (prepare to implement and implement) and institutionalization (integrate into routine practice and sustain practice) [19–22]. The stages-of-change model is compatible with other theoretical frameworks for implementation research, such as the RE-AIM framework, the Consolidated Framework for Implementation Research (CFIR) and the conceptual model of evidence-based practice implementation in public service sectors [23].

## Results

### Key features of the implementation process

A description of the chronology of planning and implementation within the stages-of-change framework is given below. In the Umbi9 study, we concentrated on the first four stages in the pre-implementation and implementation phases, as the feasibility of implementation and further scale-up had to be established before we could consider the stages related to routine and sustainable practice. Table 2 contains a brief summary of how some of the more important implementation issues were addressed within this framework. All the steps did not take place in a strictly linear fashion and more than one activity may have been undertaken at the same time.

**Table 2:**
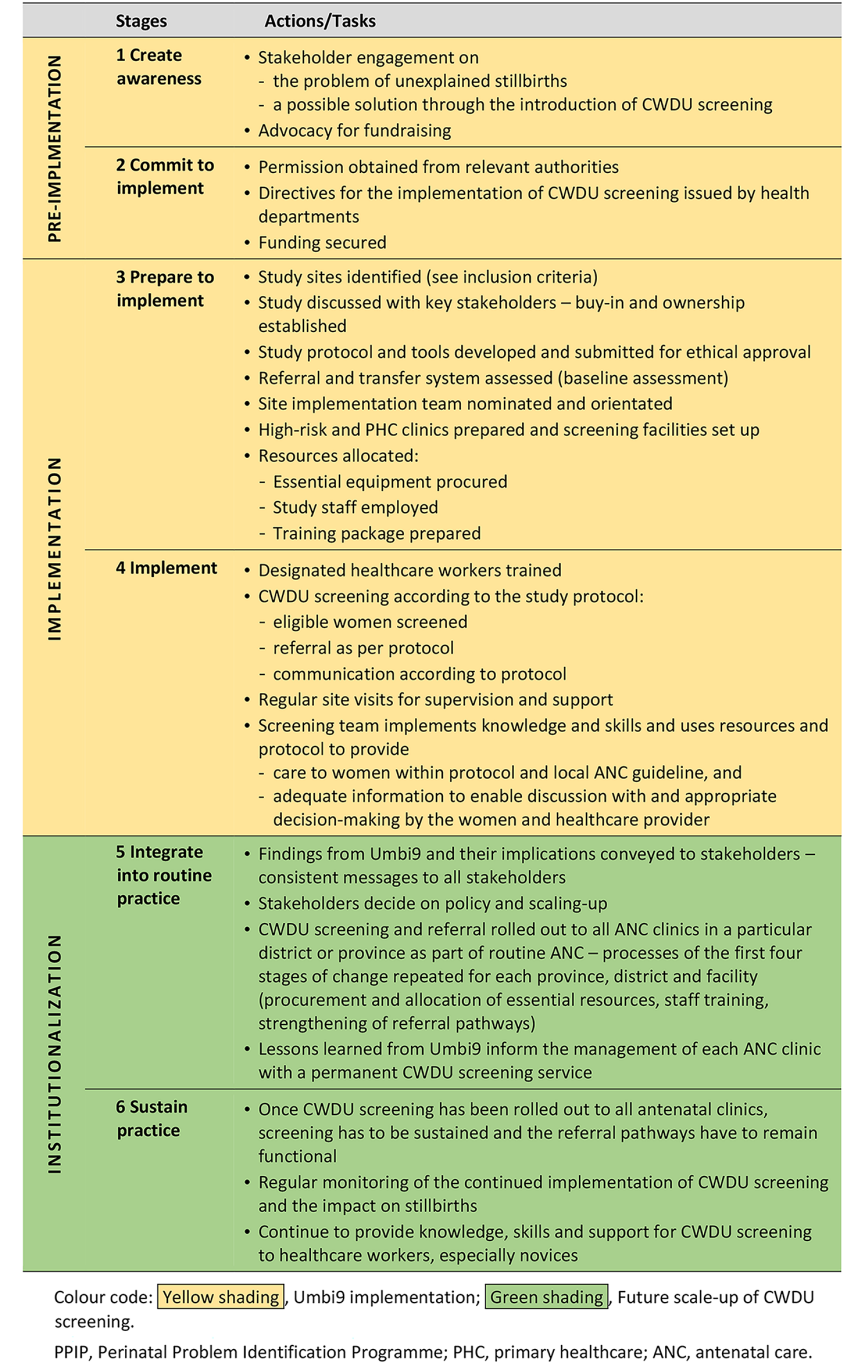
Implementation of CWDU screening according to the stages-of-change framework

### Creating awareness

A previous study on CWDU screening in Mamelodi, Pretoria, referred pregnant women in a low-risk population with abnormal placental blood flow findings (measured with the resistance index – RI) from an ANC clinic to a high-risk clinic at the regional hospital. Researchers found a higher-than-expected prevalence of abnormal RI and a significant reduction in antenatal stillbirths [12]. These findings were used to advocate for further exploration of the prevalence of abnormal RI across South Africa. The findings were shared with representatives from the national Department of Health, provincial governments, district management teams and other relevant stakeholders.

The developers of the Umbiflow™ device (SAMRC and CSIR) expressed interest in supporting further testing and rollout of the device. The University of Pretoria’s SAMRC Unit submitted a successful funding application for device testing and screening processes.

### Committing to implementation

The study had the support of the national Department of Health through the maternal, neonatal and child health programme managers, who communicated the promising results from the initial study, the CWDU screening details and a call for collaboration to the provinces. Local maternal and neonatal indicators were also used as motivators for committing to the study. Through a process of continued engagement with stakeholders such as provincial and district representatives, facility managers, hospital chief executive officers (CEOs), heads of department and healthcare providers, it was possible to get the necessary buy-in for implementing CWDU screening.

A contract was signed between the University of Pretoria as implementer and the SAMRC and CSIR as funding agencies. These agencies also provided the necessary equipment. The CSIR was responsible for Umbiflow™ probe and software development, maintenance and technical support. All healthcare services, except for the CWDU screening, were to be part of the routine activities of healthcare providers in the PHC clinics, community health centres (CHCs) and the hospitals included in the study.

### Preparing for implementation

Preparation for implementation was guided by a review of available literature and reflection on lessons from the Mamelodi study. Stakeholders were identified at the nine study sites, which included eight regional hospitals and one provincial tertiary hospital serving a mixture of urban, peri-urban and rural catchment areas. At the seven study sites with active provincial involvement, communication regarding local requirements for the study site was easier. The *engagement with local stakeholders* such as district and sub-district managers, district clinical specialist teams, managers of facilities with ANC services, hospital CEOs, clinical managers and departmental heads at each study site facilitated incorporation of the local study team in the patient pathway and ensured the necessary fluidity of recruitment and support. Table S1 in the supplementary file provides an overview of the nature of the stakeholder engagement at each of the study sites with regard to support received from district management, the district clinical specialist team, clinic and hospital management and the obstetrics and paediatrics departments.

**Fig. 2:**
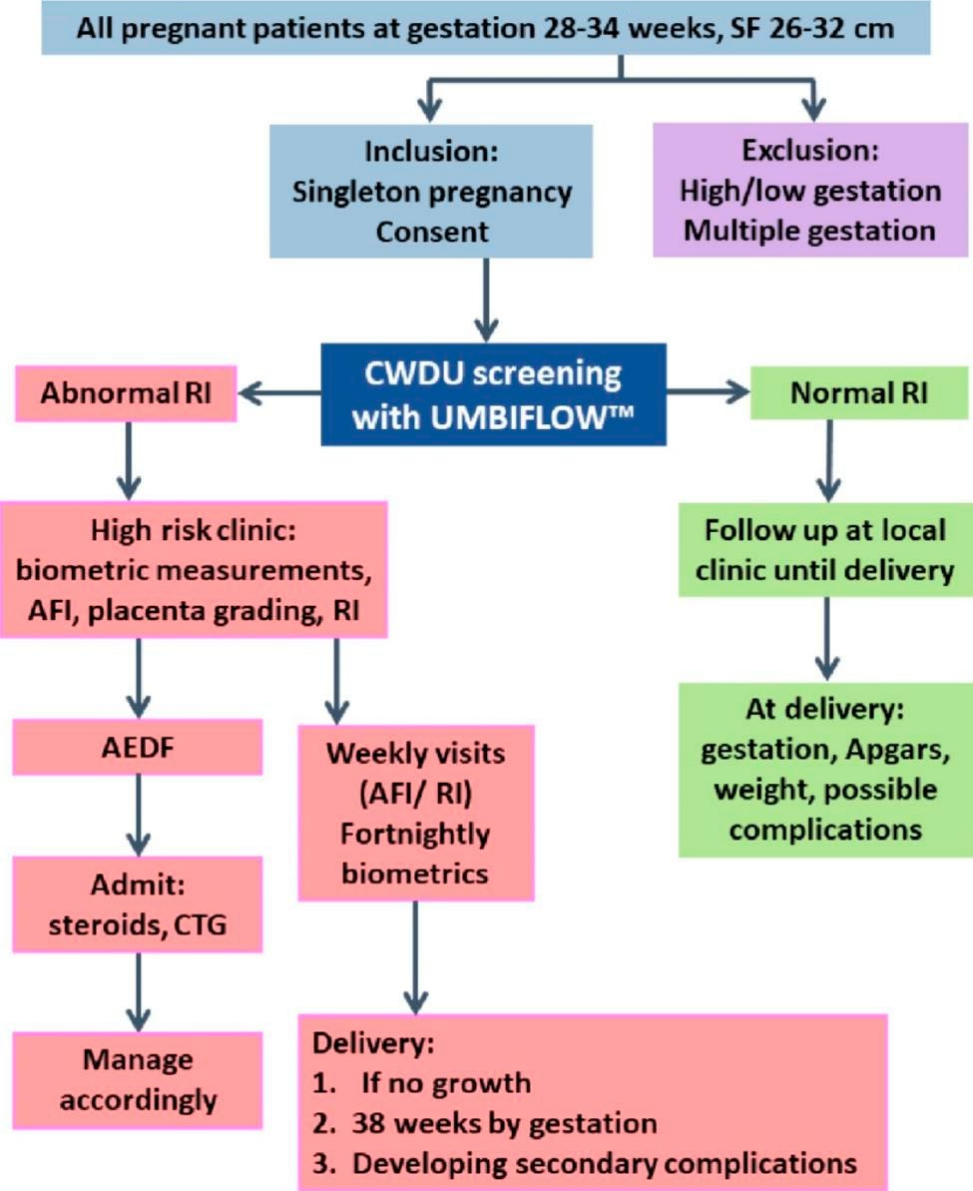
Flow diagram of participants in the study protocol SF, symphysis fundal height; CWDU, continuous-wave Doppler ultrasound; RI, resistance index; AFI, amniotic fluid index; AEDF, absent end diastolic flow; CTG, cardiotocograph

Developing the *study protocol and tools* was critical for fidelity of implementation and uniform training. The protocol and tools supplied guidelines for identification, screening, triage, treatment, management, referral and follow-up of all screened pregnant women. Figure 2 illustrates the patient pathways and flow of participants as documented in the study protocol. The study tools included the forms and questionnaires covering eligibility, contact, demographics, patient history and lifestyle, CWDU examination findings, and the delivery forms. For women with an abnormal RI, referral and follow-up forms documented the high-risk clinic contacts.

The *CWDU training package* for the study nurses built on training for the Mamelodi study, with theoretical and practical sessions to demonstrate what we were hoping to achieve by screening a low-risk population. Theoretical presentations included detailed background information on Doppler use, BANC Plus, symphysis fundal height measurement, foetal growth restriction and referral systems between facilities. Practical sessions comprised familiarization with the Umbiflow™ software and device components and troubleshooting for software-related matters. Facility visits enabled hands-on experience and learning from peers in real time, observation of facility set-up and patient follow-up, and use of the study tools.

The central research team responsible for the execution and oversight of the research project was finalized and study staff were recruited for each site. Each study site had a local *research team* at the referral hospital consisting of an obstetrician and or medical officer, a neonatologist or paediatrician, a study nurse and a data clerk. Each site team was orientated onsite and was supported by other clinicians working in maternal and neonatal care. There were no other requirements or demands on study-site teams, as the special study nurse recruited for each site was responsible for performing the screening and would be trained in the use of the Umbiflow™ device.

The implementation of the study protocol was dependent on the *resources* provided. Each site received a set of essential equipment (Microsoft computer, two Umbiflow™ probes, thermal printer, and sonar gel). The study nurse was responsible for storing the equipment and moving the items to whichever clinic was scheduled for screening on a particular day. Each referral hospital had commercial ultrasound equipment with a pulsed-wave Doppler function, a theatre(s), a blood bank, a laboratory unit, and maternity and neonatal units. There were no other additional equipment needs.

### Implementing screening

CWDU screening was implemented stepwise, one study site at a time. The sequence of implementation was purposive, according to site readiness and stakeholder responsiveness. Table S4 in the supplementary file provides an overview of the timeline of implementation. The study nurse and data clerk from each site had a week of Umbiflow™ *training*, followed by a weeklong visit to an ongoing recruiting site where they were involved with recruitment and screening. The first four site teams were trained at the original Mamelodi study site, and the next five teams did their practical training at the first implementation site of the Umbi9 study.

After training, each screening team (nurse and data clerk) returned to its site with an *initiation plan* that addressed recruitment days at ANC clinics and follow-up days at the referral hospital’s high-risk clinic. This included site plans showing where to recruit and where the study team would be stationed within the recruitment facilities. Each site had a visit from and remote sessions with the central research team to review referral routes and transfer systems and to assess feasibility, staffing, flow plan and space, and determine how CWDU screening would fit in with the patient flow through the health system. The data clerk at each site recorded all the births at CHCs, midwife-run obstetric units (MOUs) and hospitals in the catchment area of the study site. This allowed the tracking of all deliveries in the catchment areas on an electronic birth register.

*CWDU screening* occurred according to the study protocol (Fig. 2). Low-risk women presenting for antenatal care at the PHC clinics between 28 and 34 weeks’ gestation were eligible for screening. Women with a normal RI continued their antenatal care at the local clinic. Women with an abnormal RI were referred to the high-risk clinic at the referral hospital for further clinical evaluation and follow-up contacts, following a standard management protocol.

Women failing to attend the referral high-risk clinic or their next of kin were contacted by the study nurse by telephone to ascertain the reasons for their inability to attend and to arrange another contact appointment. Women contacted on two occasions at different times of the day without any response were considered lost to follow-up. High-risk clinic attendance was fair, with a loss to follow-up of 0–31% of women with an abnormal RI [13, 14]. Table 3 provides an overview of high-risk clinic attendance at the different study sites.

**Table 3:** Number of women with abnormal RI who attended high-risk clinic referral

	Recruited	Normal RI	Abnormal RI	AEDF	Abnormal RI attended HR clinic
Site	(N = 7088) n	(N = 6169) n (%)	(N = 919) n (%)	(N = 87) n (%)	(N = 730) n (%)
A	1111	883 (79.5)	228 (20.5)	10 (0.90)	157 (69.0)
B	509	467 (91.7)	42 (8.3)	6 (1.18)	32 (76.2)
C	476	449 (94.3)	27 (5.7)	4 (0.84)	22 (81.5)
D	673	616 (91.5)	57 (8.5)	7 (1.04)	52 (91.2)
E	629	520 (82.7)	109 (17.3)	9 (1.43)	98 (89.9)
F	1097	972 (88.6)	125 (11.4)	10 (0.9)	125 (100)
G	982	919 (93.6)	63 (6.4)	14 (1.4)	47 (74.6)
H	749	582 (77.7)	167 (22.3)	18 (2.4)	107 (64.1)
I	862	761 (88.3)	101 (11.7)	9 (1.0)	90 (89.1)
**Total**	**7088**	**6169 (87.0)**	**919 (13.0)**	**87 (1.2)**	**730 (79.4)**

RI, resistance index; AEDF, absent end diastolic flow; HR, high-risk

### Implementation at the individual sites

#### Primary healthcare antenatal clinics

All study sites recruited on multiple days of the week and devoted one day to a high-risk clinic at the referral hospital. Eight sites alternated between different clinics on different days of the week. Six study sites recruited participants at two clinics and two sites at three clinics on different days of the week; one site had only one clinic and recruited there on four days of the week. Alternating between different recruitment clinics affected recruitment numbers, as some clinics were busier on certain days of the week and we could not achieve 100% recruitment at all the sites. Some PHC clinics had their contacts for first pregnancy bookings on certain days of the week and CWDU screening on those days did not recruit a lot of women if they had booked early. Some late bookers may also have been missed. At clinics where screening was not integrated into the antenatal contact, some women may not have been offered the screening or may not have been willing to wait for the consultation.

Study sites considered their local circumstances when determining the day of the week on which to run the high-risk clinic. Wednesday was the most popular day, with only one site choosing Fridays. One study site started using Mondays but also switched to Wednesdays during implementation. Four hospitals saw women with abnormal RIs during the usual high-risk antenatal clinic, four had dedicated abnormal RI clinics and one hospital started with a dedicated clinic but later changed to the usual high-risk clinic model.

All clinics at the nine study sites held morning antenatal talks with all the pregnant women attending the clinic that day, followed by one-on-one discussions with the women to check for eligibility for recruitment and screening. The in-house nursing staff then referred eligible women to the study nurse. The recruitment clinics at two study sites were able to integrate CWDU screening into the routine antenatal care consultation, where a woman was assessed and had her CWDU screening during the same consultation with the study nurse. This arrangement was well received by facility staff, as the interaction between the study and facility teams improved staff morale and encouraged mutual support with patient care, as well as allowing for better patient flow and reducing waiting and consultation times. Three study sites held the CWDU examination in the same room but with a different provider. The remaining four study sites held the CWDU examinations after the antenatal consultation but in a different room.

#### High-risk hospital clinics and follow-up

The majority of clinics and hospitals were close to each other, which facilitated high-risk patient follow-up and meant decreased patient costs for follow-up. The follow-up of women with abnormal RIs was challenged by issues such as other social responsibilities, transport availability and out-of-pocket expenses incurred in reaching the hospital. In rural areas, greater distances between some PHC clinics and the hospital might have impeded patient follow-up, especially if public transport was scarce and no routine patient transport was available. All the study sites recorded better high-risk follow-up rates from nearby clinics. Five clinics were within a radius of < 5 km from the hospital and 10 were 5–10 km away. The remaining four clinics were 11, 12, 35 and 41 km from the hospital, respectively.

The 100% follow-up rate achieved at Site F was due to the patient transport made available from the remote CHC to the hospital. The hospital also had an onsite sonographer performing sonars for their high-risk women, followed by a consultation with the specialists. Regular outreach at Site I from the hospital’s obstetrics unit to PHC clinics contributed to women’s acceptance of the importance of follow-up. It served as a motivating factor because women got to know the doctors at the hospital. At the other two study sites with a follow-up rate of more than 89% (Sites D and E), it was observed that the study nurses had a very good rapport with the clients. The four study sites with the highest high-risk follow-up rate had employed an advanced midwife as study nurse.

#### Continuous communication and site visits

Monitoring and evaluation was enabled by regular communication between the central research team and the site teams (see Fig. 1). The principal researcher also conducted site visits, once in the first quarter of implementation and thereafter six-monthly. During the first site visit, progress with the study, recruitment, probe use, the screening process, and the screening results were reviewed and other challenges addressed. The referral and follow-up of women identified as having abnormal RIs were assessed and, where available at the time of the visit, the management protocol at the high-risk clinics and outcomes were also reviewed. At all study sites time was spent on reviewing the Doppler waveforms and attending to waveform capturing, management plans and further troubleshooting. The review of the study files included checking the correct completion of all files, the details on the high-risk clinic forms, and the entry of delivery outcomes. Progress with data capturing on the electronic birth register was also reviewed. Completed study files were collected for collation at the SAMRC Unit for data entry on RedCap software.

Continuous communication and site visits facilitated tasks, actions, and decision-making during the training and implementation period and allowed for adjustments for unexpected challenges. Sites reported the same challenges during remote support and onsite visits. With regard to recruitment, two sites had difficulty in identifying women eligible for screening on the screening day. The study nurses were able to move to another recruitment clinic for that day or to cover two clinics on the same day. At six sites national and provincial service protests jeopardized the safety of patients and staff, and disrupted recruitment and screening to varying degrees. At such times recruitment was halted or alternative arrangements were considered for screening and follow-up, for example continued screening in areas where there were no protests, continuing the high-risk clinic if the hospital was accessible, and telephonically checking up on women for alternative arrangements for their follow-up date. Two sites also reported challenges with follow-up at the high-risk clinic because of the unavailability of the head of department, who had relocated; however, the reviews at these clinics were able to continue with the available medical officers and the orientation of the new specialists. One site also had to deal with the logistics of the relocation of a clinic site due to renovations and at one site the replacement of the study nurse led to a break in recruitment. Unforeseen delays in the commencement of CWDU screening at some sites were the result of late approval from one provincial research committee and difficulties in setting up the screening space at two clinics.

## Discussion

The Umbi9 study was successful with regard to the implementation of screening according to protocol and in terms of the outcomes in the reduction of stillbirths [13, 14]. In this section we reflect on the lessons learned through the implementation of CWDU screening, how to integrate the screening into routine antenatal care and how to select the most appropriate CWDU screening models for scale-up.

### Lessons learnt

Lessons learnt from the Umbi9 implementation study will be valuable for future studies and for the scale-up of CWDU screening. Table 4 provides a brief summary of some of the important lessons.

**Table 4:** Important lessons from the Umbi9 implementation study

Lesson	Implications for further scale-up
1. Stakeholder involvement and continuous communication	• Ensure that all key stakeholders are engaged in the planning and rollout of the intervention, including the use of community platforms (e.g. community leaders, hospital groups, church groups, local support groups, MomConnect), pamphlets and radio • Assists with acceptance and better integration, especially in the early stages of implementation • Encourages and strengthens facility engagements and communication between management and facility staff
2. Recruiting and screening at PHC clinic level	• Clinic healthcare providers feel empowered • Enhanced staff responsiveness to monitoring foetal growth and assessing for placental insufficiency • Heightened awareness of importance of recognizing pregnancy at risk – staff more inclined to consult with each other about patients with complications
3. Integrating CWDU screening into routine work	• Staff should be trained and accept the intervention • Staffing, resources and arrangement of staff duties should be considered and reorganized • Daily screening is needed to ensure the coverage of all pregnant women • Outreach doctors could evaluate placental function at the clinic if pulsed-wave ultrasound is available at PHC clinics • Effective data collection and monitoring tools are essential to reduce the burden on staff and assist with swift adoption in the health system
4. Training and learning when CWDU screening is introduced	• There is a fast learning curve, with nursing teams able to use the probe after a short period of training • Reassurance can be given that other staff would be able to use the Umbiflow™ device • Peer learning occurs through observation of others performing the examination • Onsite training allows for better learning • Greater staff awareness of antenatal and postpartum needs in the screened women is created
5. Communication	• Continuous communication with the study and implementation teams allows for timeous solutions and planning of next steps • Virtual communication allows for real-time access to data and information and allows for troubleshooting issues to be solved timeously • Good rapport between the referring nurse and patients may contribute to better follow-up rates at the high-risk clinic – advanced midwives are familiar with antenatal care and medium and high-risk obstetric care and would be able to reassure women and help them prepare for the pregnancy journey and experience
6. Site support visits	• Continuous stakeholder engagement is enabled • Data collection, data entry, recruiting and screening challenges can be addressed and timeous solutions can be found • Reassurance is given that implementation progresses as planned
7. Referral after CWDU screening	• Improvement of the referral pathways and communication between the different levels of care could be facilitated • Available patient transport facilitates women’s attendance of the high-risk antenatal clinic • Open communication pathways, a functional referral system and transport assist screening and the further management of women identified with abnormal RIs • Outreach needs and possibilities should be considered • Continuum of care could be enhanced in urban areas with mobile populations by ensuring that pregnant women who move from one area to another or who ask to be referred to a different hospital receive a detailed referral letter (including the Doppler findings) to enable the next health team to manage these women appropriately
8. Acceptance of patients at the referral hospitals	• Because referral hospitals see the importance of screening, acceptance of patients at the high-risk hospital clinics is perhaps not as big a factor as previously thought • Each referral hospital must establish a high-risk antenatal clinic for women with abnormal RIs, or reorganize ANC to incorporate women identified with abnormal RIs in the current high-risk clinic(s) at the hospital • Neonatal services should be prepared for challenges related to admissions of CWDU-screened neonates
9. Robust and durable equipment	• Umbiflow™ equipment set could be moved daily from clinic to clinic – able to withstand a lot of handling and proved to be low on maintenance • Only two high-volume sites required replacement of the probe cord – new upgraded probes are cordless and allow for even better mobility and accessibility • The small thermal printers did not need any ink and did not require frequent paper changes

PHC; primary healthcare; RI, resistance index; ANC, antenatal care

#### Integrating and sustaining CWDU screening in routine antenatal care

For the Umbi9 study, specially trained nurses performed the screening examinations at the study sites. They were an additional human resource who helped share the daily clinical patient load. With any further rollout the screening should be done by the nurses and doctors who are already in the system. What remains to be determined is the acceptability of integrating CWDU screening into routine antenatal care services *without additional staff and resources*. Of particular importance is the initial training of antenatal nurses and doctors who would be responsible for conducting the CWDU screening. These issues could be investigated from a supply- and demand-side perspective, and costs should be looked at. Aspects to consider include additional time needed at the 30-week contact for both staff and clients and the influence on waiting times. The ease of use of the Umbiflow™ device by nurses also needs further confirmation and training and equipment must be budgeted for.

The *unique circumstances of each screening catchment site* need to be constantly evaluated to address challenges and find feasible solutions. The influence of service protests on recruitment and follow-up is one such example. Furthermore, changes in patient flow within the health system could affect implementation and require consideration. Finding solutions to challenges should be a priority in order to promote implementation and future sustainability of CWDU screening.

The Umbi9 study only completed the first four stages of the six stages of change illustrated in Table 1. For scaling up, CWDU screening needs to be *integrated into routine antenatal care services* (Table 1, Stage 5). Once CWDU screening has been rolled out to all antenatal clinics in a particular administrative area, screening has to be *sustained* and the referral pathways must remain functional (see Table 1, Stage 6). For successful implementation the processes of the first four stages of change have to be repeated for each province, district and facility, and health system requirements needed to sustain and integrate screening and management of women identified with abnormal RIs should be attended to. CWDU equipment should be placed on essential equipment lists. Regular monitoring of the continued implementation of CWDU screening and the impact on stillbirths is also essential.

#### Models for implementing and scaling up CWDU screening

**Fig. 3:**
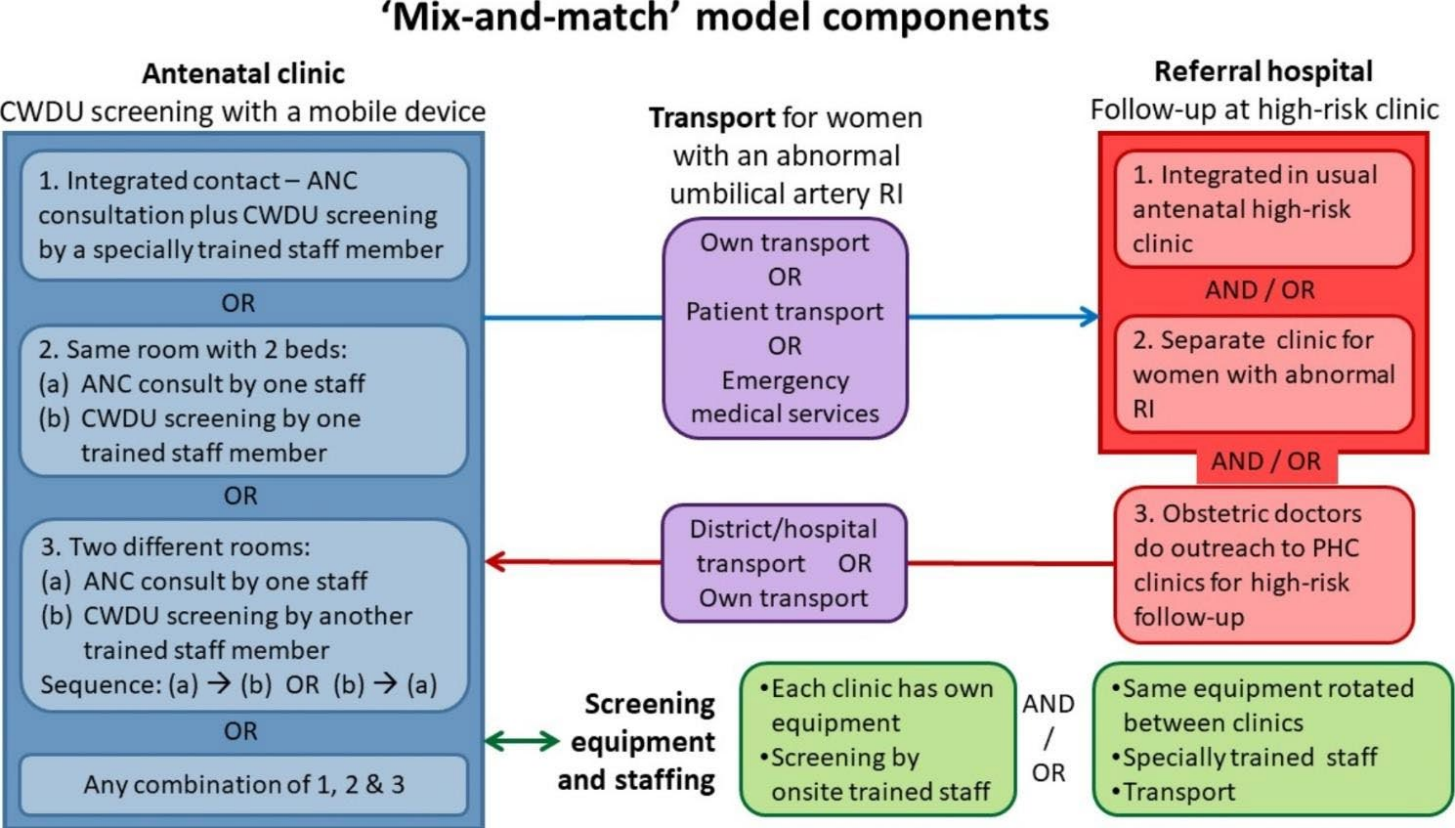
Components for building implementation models for CWDU screening CDWU, continuous-wave Doppler ultrasound; ANC, antenatal care; RI, resistance index; PHC, primary healthcare

In future, flexible, contextually-responsive ‘models’ of CWDU screening implementation should be explored to enable a smooth transition to routine practice – a one-size-fits-all model does not work. Policymakers and implementers in an administrative or geographic area should decide on the contextually most appropriate model or models for the implementation of CWDU screening, based on the unique circumstances of each catchment area and the availability of screening, referral and transport in the area. Figure 3 presents the various implementation components that emanated from the Umbi9 study. This model may also be applicable to the implementation of other types of mobile equipment in PHC services. In the case of CWDU screening, there are four components to consider:

1. The way CWDU screening will be done during the antenatal contact at each individual clinic earmarked for CWDU services (blue).
2. The way in which the follow-up of women with abnormal RIs will be accommodated in a high-risk clinic at the referral hospital (red).
3. Transport arrangements to facilitate access for women with abnormal RIs to high-risk antenatal care and treatment at the referral hospital (purple).
4. How the CWDU equipment will be distributed and managed between clinics and implications for staffing requirements (green).

At the antenatal clinic, the clinic set-up may determine the patient flow (blue blocks in Fig. 3). The ideal would be to have one point of contact for the ANC consultation and the CWDU screening by the same staff member, who has been trained in the application of CWDU. It would be preferable to have at least two staff members trained in CWDU to cover for absences when one of the two is on leave. Other options are having the consultations and the screening in the same room, but done by different staff members, or having the two activities in two different rooms. In high-volume clinics with only one CWDU-trained person, one of the last two options may have to be considered. Waiting times need to be considered in future implementation work when women have to wait for their antenatal contact and then again for the screening.

Another choice pertains to the CWDU equipment and staffing for screening (green blocks in Fig. 3). The ideal would be for each clinic to have its own equipment and have staff trained in CWDU screening. Where that is not possible, mobile equipment with specially trained nurses who rotate between clinics could be considered. A potential advantage of having onsite screening equipment is higher coverage of women being screened and implicitly better attendance of the high-risk clinic, but this requires training of large numbers of staff in order to have a nursing team at each clinic to screen women daily. A model with mobile equipment and rotation between clinics might increase accessibility and reduce equipment costs, but would require additional staff and transport for travel between clinics.

The implementation model includes considering what would be the most efficient referral and follow-up systems (red blocks in Fig. 3). Screened women with an abnormal RI need to be referred to the appropriate next level of care, which may be a regional, provincial tertiary or central hospital. At the referral hospital these women could be seen during the usual high-risk antenatal care clinic, which would mean that existing work and patient flows could be maintained. The second option would be a separate clinic with its own time slot at the hospital, which may be a practical or acceptable option for hospitals with large volumes of patients attending the usual high-risk antenatal clinic. A third option would be for doctors at the hospital’s obstetric unit to follow up high-risk women close to their homes through their outreach activities to PHC facilities. Although this may allow for close to 100% follow-up of women and less cost to the patient, this may not be practical in the case of clinics with low antenatal bookings.

Accessibility to high-risk antenatal follow-up is key for women with an abnormal RI. Transport to and from clinics to the referral hospital is a crucial factor that affects follow-up (purple blocks in Fig. 3). In the Umbi9 study sites, women cited transport costs as a reason for not being able to attend hospital follow-up. Therefore, transport options should be worked out to ensure that no woman is left behind in getting the necessary high-risk antenatal care. Feasible solutions such as patient transport, involving emergency medical services, and outreach services as mentioned above should be considered to alleviate the financial burden on women.

#### Limitations

Although the insights gleaned from this implementation study may not be generalizable to all hospitals, ANC clinics and health catchment areas in South Africa, the choice of sites in eight of the nine provinces in South Africa was an attempt to include diversity in site selection, as provinces are governed in different ways. The purposive sample of sites that fulfilled all the inclusion criteria potentially described an ideal situation. We could not assess the real effect on the neonatal services as we knew the neonatal services were adequate to start with. A next step underway is the identification of barriers and enablers to implementation of screening in the Tshwane health district in South Africa. In terms of the implementation of CWDU screening, we could not describe all six stages in the stages-of-change framework as Umbi9 was a pilot study. A longer-term focus would be further scale-up and monitoring of the sustainability of the screening. A further limitation was the employment of two additional staff members to implement the screening at each study site, one to do the screening and one for data collection and collation. It is not known how staffing might be affected once CWDU screening is integrated into routine antenatal care without additional staff. We were also unable to test the different implementation models depicted in Fig. 3 and calculate the cost of each model; for this a subsequent study would be needed.

## Conclusion

This study has shown that introducing CWDU screening with appropriate follow-up is feasible in a middle-income country and results in a step-change reduction in stillbirths. Preventing stillbirths requires commitment from healthcare providers and costs money, even with low-cost equipment. If the ‘neglected tragedy’ of 2 million stillbirths per year in mainly LMICs [24] is to be taken seriously, then implementing feasible interventions that have demonstrated a major impact on stillbirth rates should receive the necessary investment.

## Electronic supplementary material

Below is the link to the electronic supplementary material.

**Additional file 1**: Supplementary Tables

## Data Availability

All data generated or analysed during this study are included in this published article and its supplementary information files.

## Abbreviations

AEDF: Absent end diastolic flow
AFI: Amniotic fluid index
ANC: Antenatal care
BANC: Basic Antenatal Care
CHC: Community health centre
CSIR: Council for Scientific and Industrial Research
CTG: Cardiotocograph
CWDU: Continuous-wave Doppler ultrasound
HR: High risk
LMIC: Low- and middle-income country
MOU: Midwife-run obstetric unit
PHC: Primary healthcare
PPIP: Perinatal Problem Identification Programme
RH: Regional hospital
RI: Resistance index
SAMRC: South African Medical Research Council
SF: Symphysis fundal height
WHO: World Health Organization
