# Supplementary material for: Implementation of continuous-wave Doppler ultrasound to detect the high-risk foetus in the low-risk mother: lessons from South Africa
Source: BMC Pregnancy Childbirth. 2023 May 27;23:393. doi: 10.1186/s12884-023-05721-3 (PMC10223844; doi:10.1186/s12884-023-05721-3)
Supplement: Supplementary file 1 — Additional file 1: Supplementary Tables [file 12884_2023_5721_MOESM1_ESM.pdf]

SUPPLEMENTARY MATERIALS

Content

TABLE S1. Overview of implementation at individual study sites 1

TABLE S2. Umbi9 recruiting primary healthcare clinic characteristics 11

TABLE S3. Umbi9 referral hospitals: inclusion criteria fulfilled 13

TABLE S4. Overview of activities of the central research team and the local study teams across the nine sites 15

TABLE S1. Overview of implementation at individual study sites

Abbreviations:

ANC	Antenatal Care
BANC	Basic Antenatal Care
CEO	Chief Executive Officer
CHC	Community Health Centre
DCST	District Clinical Specialist Team
HOD	Head of Department
MOU	Midwife-led Obstetric Unit
RI	Resistance Index
SAMRC	South African Medical Research Council
UP	University of Pretoria

**Characteristics and processes**	**Site-specific description**
**Site A**	
Stakeholder engagement:	• Meeting with Hospital CEO, Head of Department (HOD) and management<br>• Meeting with clinics and clinicians<br>• Meeting with combined management, including DCST and district representatives on BANC Plus and Umbiflow™
Setting up the facilities:	• Hospital A also runs an ANC clinic for low-risk women based on geographic boundaries and was included as a recruitment site<br>• Clinic 1 (MOU) was included based on referral patterns and PPIP data reports<br>• Space identified at both recruitment sites
Composition of onsite study team:	• Study nurse, data clerk, hospital doctors<br>• Hospital team: HOD and sonographer
Training of study nurse and data clerk:	• Training in Pretoria (10 days)<br>• Practical at Mamelodi study site (site in previous study)<br>• Electronic birth register data entry
Clinic arrangements for Umbiflow™ screening:	• Clinic 1: separate consultation room dedicated for Umbiflow™ screening<br>• Hospital A: small space created to labour ward for Umbiflow™ screening and for high-risk patient follow-up (room with bed and table for screening and sonar for the women with abnormal RIs)
Recruitment schedule:	• Tuesday-Friday: Clinic 1<br>• Monday: Hospital A low-risk ANC clinic
High-risk clinic:	• Initially Mondays, run by HOD and sonographer<br>• Changed to Wednesday to coincide with support at usual high risk-clinic (UP/SAMRC Unit research staff visits on Wednesday)

Characteristics and processes	Site-specific description
Issues raised during virtual support:	• Digital Umbiflow™ interface:<br>- Testing software settings<br>- Adjusting the probe sound<br>• Stationary and equipment needs (printing paper, printer cable, study booklets, referral forms, delivery forms)
Issues raised during onsite visits:	• Recruitment challenges (number of eligible women on specific days of the week; national protests making facilities inaccessible due to safety reasons for staff and patients)<br>• Review of the study files (check that all forms are filled in correctly, high-risk clinic forms are detailed and delivery outcomes are followed up)<br>• Progress with implementation of electronic birth register<br>• Most raised issue: Waveform capturing (checking the Doppler waveforms and management plans)
Unforeseen circumstances and other observations:	• Delays in setting up the site and procuring equipment, resulting in a recruitment delay of 3 weeks<br>• Sonographer involvement on high-risk clinic days<br>• Provincial, district and DCST not involved in team work
**Site B**	
Stakeholder engagement:	• Site discussions with Province, District, Hospital management, clinics, DCST, clinicians<br>• Meeting with hospital CEO, HOD and management<br>• Meeting with clinics and clinicians discussing BANC Plus and Umbiflow™<br>• Meeting with combined management, including DCST and district representatives for BANC Plus and Umbiflow™
Setting up the facilities:	• Based on the PPIP data findings and unique circumstances of this area, 3 ANC clinics were identified for screening on different days of the week<br>• Small town and all patients deliver at the hospital irrespective of their risk category<br>• At each clinic a dedicated Umbiflow™ consultation room where Umbiflow™ screening and the antenatal consultation done together by Umbiflow™ nurse
Composition of onsite study team:	• Study nurse, data clerk, hospital doctors<br>• Hospital team: HOD and two medical officers
Training of study nurse and data clerk:	• Training in Pretoria (10 days)<br>• Practical at Mamelodi study site<br>• Electronic birth registers data entry
Clinic arrangements for Umbiflow™ screening:	• Screening and recruiting integrated into ANC contact/consultation<br>• A consultation allocated to the study nurse<br>• Umbiflow™ screening and antenatal consultation done by the study nurse at all three clinics
Recruitment schedule:	• Monday: Clinic 1<br>• Tuesday & Friday: Clinic 2<br>• Thursday: Clinic 3
High-risk clinic:	• Wednesday, run by HOD and 2 medical officers

Characteristics and processes	Site-specific description
Issues raised during virtual support:	• Digital Umbiflow™ interface (testing software settings and adjusting the probe sound) – study nurse and data clerk able to remotely solve most of the technical issues<br>• Stationary and equipment needs (printing paper, printer cable, study booklets, referral forms, and delivery forms)<br>• Abnormal RI women and follow-up challenges (HOD on extended leave for 6 months, medical officers continued running the high-risk clinic)<br>• How to address change in clinic location due to ongoing renovations.<br>• New clinic venue was a community hall with partitions for ANC consultations
Issues raised during onsite visits:	• Flexibility to visit more than one clinic when the numbers are low at one clinic.<br>• Clinic 3 further from hospital on opposite side of town and more peri-urban and rural than the other 2 clinics<br>• Recruitment challenges on certain days of the week. If no eligible women, the study nurse was able to go to another clinic (this was promoted by good communication between study nurse and the clinic staff)<br>• Study files review of data entry, tracing women lost to follow up.<br>• Progress with implementation of electronic birth register<br>• Waveform capturing and saving the Umbiflow™ tracing
Unforeseen circumstances and other observations:	• Clinic 1 had renovation mid-year and the clinic was relocated to local community hall – study team able to move with clinic and continue recruiting<br>• DCST actively involved, encouraged the study team and helped with BANC Plus support
**Site C**	
Stakeholder engagement:	• Site discussions with Province, District, Hospital management, clinics, DCST, clinicians<br>• District team meeting to discuss Umbiflow™ and suitable clinics for this initiative<br>• Clinic visits with District management for site view and introductions at clinics<br>• Meeting with hospital CEO, HOD and management – invited to hospital CEO office for discussion and approvals<br>• Meeting with clinics and clinicians discussing BANC Plus and Umbiflow™
Setting up the facilities:	• Clinic 1 has an ANC clinic container with one room and 2 beds<br>• Clinic 2 has space available – dedicated Umbiflow™ room
Composition of onsite study team:	• Study nurse, data clerk, hospital doctors<br>• Hospital team: one specialist and one medical officer
Training of study nurse and data clerk:	• Training in Pretoria (10 days)<br>• Practical at Mamelodi study site<br>• Electronic birth register data entry
Clinic arrangements for Umbiflow™ screening:	• Clinic 1: ANC in clinic container – consultation room with 2 beds partitioned for privacy – women have ANC consult, and if illegible, are offered Umbiflow™ screening on other side of room<br>• Clinic 2 had space available, with a consultation room dedicated for Umbiflow™ screening on specific days of the week

Characteristics and processes	Site-specific description
Recruitment schedule:	• Monday and Tuesday: Clinic 1<br>• Thursday and Friday: Clinic 2
High-risk clinic:	• Wednesday, run by specialist and medical officer
Issues raised during virtual support:	• Service provision protest disrupting recruiting and screening<br>• Digital Umbiflow™ interface (testing software settings and adjusting the probe sound) – study nurse and data clerk able to remotely solve most of the technical issues<br>• Stationary and equipment needs (printing paper, printer cable, study booklets, referral forms, and delivery forms)
Issues raised during onsite visits:	• Clinic issues related to access during protest times<br>• No clinic and hospital access during protest times. Hospital was more accessible, and high-risk clinics continued, but local PHC clinics were closed due to safety issues.<br>• Study files (review; and equipment and stationary audits).<br>• Delivery of equipment and stationary as per the study needs.<br>• Progress with implementation of electronic birth register<br>• Waveform capturing (checking the Doppler waveforms and management plans)
Unforeseen circumstances and other observations:	• Study team adjustments when the initial high-risk clinic specialist relocated to a different hospital – joined by new specialist – support visit for new team member to actively take over the role in the high-risk clinic<br>• Province severely affected by service provision for over 3 months protests with no safe access to health care facilities – no recruitment during protest months
**Site D**	
Stakeholder engagement:	• Site discussions with Province, District, Hospital management, clinics, DCST, HOD, clinicians<br>• District team meeting to discuss Umbiflow™ and suitable clinics for this initiative<br>• Clinic visits with District management for site view and introductions at the clinics<br>• Meeting with hospital CEO, HOD and management<br>• Meeting with clinics and clinicians discussing BANC Plus and Umbiflow™<br>• District formally involved in recruiting and appointing study team members (study nurse and data clerk) – district advertisement, district shortlisting and interview for both individuals
Setting up the facilities:	• Only one recruitment site – one CHC (Clinic 1)<br>• Clinic 1 has a data clerk room available<br>• Umbiflow™ recruiting and screening in same room as ANC consultation<br>• Clinic consultation has a large room with two beds, one for the ANC consultation and another for Umbiflow™ screening – partition available for privacy
Composition of onsite study team:	• Study nurse, data clerk, hospital doctors<br>• Hospital team: one specialist and one medical officer
Training of study nurse and data clerk:	• Training in Pretoria (10 days)<br>• Practical at Mamelodi study site<br>• Electronic birth register data entry

Characteristics and processes	Site-specific description
Clinic arrangements for Umbiflow™ screening:	• Umbiflow™ recruiting and screening in the same room<br>• Clinic consultation has a large room with two beds, one for the ANC consultation and another for Umbiflow™ screening – partition for privacy
Recruitment schedule:	• Monday, Tuesday, Thursday & Friday: Clinic 1<br>• Study nurse worked with clinic staff in same room – clinic staff did ANC consultation and study nurse did Umbiflow™ screening in a partitioned section of the room
High-risk clinic:	• Wednesday, run by the medical officer<br>• Umbiflow™ high-risk clinic integrated into main hospital high-risk clinic – same day and setting
Issues raised during virtual support:	• Digital Umbiflow™ interface:<br>- Testing software settings<br>- Adjusting the probe sound<br>• Stationary and equipment needs (printing paper, printer cable, study booklets, referral forms, and delivery forms)
Issues raised during onsite visits:	• Recruitment challenges during the national protests<br>• Study files data entry and Doppler waveform review<br>• Progress with implementation of electronic birth register<br>• Waveform capturing (reviewing the Doppler waveforms and management plans)<br>• Delivery of equipment and study files
Other observations and unforeseen circumstances:	• Obstetric specialist relocated to the private sector
**Site E**	
Stakeholder engagement:	• Site discussions with Province, District, Hospital management, clinics, DCST, HOD, clinicians.<br>• District team meeting to discuss Umbiflow™ and suitable clinics for this initiative<br>• Clinic visits with District management for site view and introductions at the clinics<br>• Meeting with hospital CEO, HOD and management<br>• Meeting with clinics and clinicians discussing BANC Plus and Umbiflow™<br>• Meeting with clinicians and HOD – discussions on Umbiflow™ and BANC Plus<br>• DCST and Hospital management actively involved in study team recruitment
Setting up the facilities:	• Three clinics reviewed for possible inclusion – two included based on feasibility and clinic space<br>• Clinics had a dedicated Umbiflow™ consultation room with ANC consultation in separate room
Composition of onsite study team:	• Study nurse, data clerk, hospital doctors<br>• Hospital team: HOD and medical officers
Training of study nurse and data clerk:	• Training in Pretoria (10 days)<br>• Practical at Study site A<br>• Electronic birth register data entry
Recruitment schedule:	• Monday and Tuesday: Clinic 1<br>• Wednesday and Thursday: Clinic 2

Characteristics and processes	Site-specific description
High-risk clinic:	• Wednesday, a dedicated abnormal RI clinic<br>• Run by HOD and medical officers
Clinic arrangements for Umbiflow™ screening	• Dedicated Umbiflow™ consultation room at both clinics<br>• Study nurse and facility staff worked together and when clinic was short staffed study nurse did Umbiflow™ and ANC consultation at the same setting
Issues raised during virtual support:	• Digital Umbiflow™ interface:<br>- Testing software settings<br>- Adjusting the probe sound<br>• Stationary and equipment needs (printing paper, printer cable, study booklets, referral forms, and delivery forms)<br>• Recruiting numbers and referral pattern<br>• Challenges with high-risk clinic if HOD not available for clinic – medical officers available to review patients – study support of HOD
Issues raised during onsite visits:	• Recruitment challenges during the national protests<br>• Study files data entry and Doppler waveform review<br>• Progress with implementation of electronic birth register, three hospitals for delivery outcomes<br>• Waveform capturing and review waveforms<br>• Delivery of equipment and study files<br>• High-risk clinic support<br>• Collecting completed study files for collation
Other observations and unforeseen circumstances:	• Change of HOD during recruiting time – new head joined study team<br>• Change in high-risk clinic team, with new HOD and staff rotations<br>• Clinic 3 reviewed, but not included as a recruitment site due to space and patient flow issues<br>• Three delivery sites included for the implementation of the electronic birth register (Hospitals E, E1 and E2)
**Site F**	
Stakeholder engagement:	• Site discussions with Province, District, Hospital management, clinics, DCST, HOD, clinicians<br>• Meeting with combined management, including DCST and district representatives for BANC Plus and Umbiflow™<br>• Meeting with HOD and management<br>• Meeting with clinics, clinicians and sonographer
Setting up the facilities:	• Both clinics had dedicated Umbiflow™ screening room<br>• ANC consultation integrated with screening in same setting
Composition of onsite study team:	• Study nurse, data clerk, hospital doctors and sonographer<br>• Hospital team: HOD, specialist, medical officers and sonographer
Training of study nurse and data clerk:	• Training in Pretoria (10 days)<br>• Practical at Study site A<br>• Electronic birth register data entry
Clinic arrangements for Umbiflow™ screening:	• Study nurse reviewed pregnancy – did ANC consultation and Umbiflow™ screening together in both clinics
Recruitment schedule:	• Monday and Thursday: Clinic 1 (CHC)<br>• Tuesday and Friday: Clinic 2
Day for high-risk clinic:	• Wednesday, women seen by sonographer and doctors<br>• Clinic combined with hospital's regular high-risk clinic

Characteristics and processes	Site-specific description
Issues raised during virtual support:	• Digital Umbiflow™ interface:<br>- Testing software settings<br>- Adjusting the probe sound<br>• Stationary and equipment needs (printing paper, printer cable, study booklets, referral forms, and delivery forms)<br>• Recruiting numbers and referral pattern<br>• Probe issues that resulted in a need for a new probe delivery
Issues raised during onsite visits:	• Study files data entry and Doppler waveform review<br>• Progress with implementation of electronic birth register ad delivery outcome data entry<br>• Waveform capturing and review of waveforms<br>• Delivery of equipment and study files<br>• High-risk clinic support<br>• Taking study files back to UP/SAMRC Unit office for collation
Other observations and unforeseen circumstances:	• Sonographer involvement on high-risk clinic days<br>• Patient transport available between Clinic 1 and the hospital
**Site G**	
Stakeholder engagement:	• Site discussions with Province, District, Hospital management, clinics, DCST, HOD, clinicians<br>• District team meeting to discuss Umbiflow™ and suitable clinics for this initiative<br>• Clinic visits with District management for site view and introductions at the clinics<br>• Meeting with hospital CEO, HOD, and management<br>• Meeting with clinics and clinicians discussing BANC Plus and Umbiflow™<br>• Meeting with clinicians and HOD, discussions on Umbiflow™ and BANC Plus<br>• Hospital safety group discussion<br>• DCST actively involved with identifying feasible study nurse and data clerk to join the team
Setting up the facilities:	• Clinic 1: one room – two beds – partition for privacy<br>• Clinic 2: dedicated Umbiflow™ room
Composition of onsite study team:	• Study nurse, data clerk, hospital doctors<br>• Hospital team: HOD, specialist and medical officers
Training of study nurse and data clerk:	• Training in Pretoria (10 days)<br>• Practical at Study site A<br>• Electronic birth register data entry
Clinic arrangements for Umbiflow™ screening:	• Clinic 1: large room with ANC consultation with two beds, one for ANC consultation and another for Umbiflow™ screening – partition for privacy – after ANC consultation, women offered Umbiflow™ screening<br>• Clinic 2: dedicated Umbiflow™ room on specific days of the week
Recruitment schedule:	• Monday & Thursday: Clinic 1<br>• Wednesday &Friday: Clinic 2 (or Town Clinic)
High-risk clinic:	• Wednesday’s run by the specialist and medical officers<br>• Abnormal RI clinic incorporated in hospital’s main high-risk clinic

Characteristics and processes	Site-specific description
Issues raised during virtual support:	• Digital Umbiflow™ interface:<br>- Testing software settings<br>- Adjusting the probe sound<br>• Stationary and equipment needs (printing paper, printer cable, study booklets, referral forms, and delivery forms)<br>• Recruiting numbers and referral pattern, challenges with high-risk clinic when the team changed
Issues raised during onsite visits:	• Recruitment challenges during the national protests (North West province most affected)<br>• Study files data entry and Doppler waveform review<br>• Progress with implementation of electronic birth register, three hospitals for delivery outcomes<br>• Waveform capturing and review waveforms<br>• Delivery of equipment and study files<br>• High-risk clinic support<br>• Taking completed study files back for collation
Other observations and unforeseen circumstances:	• Clinic 2 recruitment delays due to space and other programmes at the facility – Umbiflow™ screening on days when other programmes were not at the clinic (shared space with these projects)<br>• Study nurse relocated – DCST assisted with identifying new study nurse<br>• Recruitment paused while waiting for new study nurse to complete her training
**Site H**	
Stakeholder engagement:	• Site discussions with Province, District, Hospital management, clinics, DCST, HOD, clinicians<br>• District team meeting to discuss Umbiflow™ and suitable clinics for this initiative<br>• Clinic visits with District management for site view and introductions at the clinics<br>• Meeting with hospital CEO, HOD, and management.<br>• Meeting with clinics and clinicians discussing BANC Plus and Umbiflow™<br>• DCST lead clinic visits<br>• Meeting with clinicians and HOD, discussions on Umbiflow™ and BANC Plus<br>• DCST and nursing management team actively involved with identifying study nurse and data clerk to join the team
Setting up the facilities:	• Clinics 1 and 2 had a dedicated Umbiflow™ room
Composition of onsite study team:	• Study nurse, data clerk, hospital doctors<br>• Hospital team: HOD, specialist and medical officer
Training of study nurse and data clerk:	• Training in Pretoria (10 days)<br>• Practical at Study site A<br>• Electronic birth register data entry
Clinic arrangements for Umbiflow™ screening:	• Dedicated Umbiflow™ room at both clinics
Recruitment schedule:	• Monday & Wednesday: Clinic 1<br>• Tuesday & Friday: Clinic 2
High-risk clinic:	• High-risk clinic at hospital Monday-Friday<br>• Friday dedicated for the abnormal RI clinic

Characteristics and processes	Site-specific description
Issues raised during virtual support:	• Digital Umbiflow™ interface:<br>- Testing software settings<br>- Adjusting the probe sound<br>• Stationary and equipment needs (printing paper, printer cable, study booklets, referral forms, and delivery forms)<br>• Recruiting numbers and referral pattern
Issues raised during onsite visits:	• Study files data entry and Doppler waveform review<br>• Progress with implementation of electronic birth register<br>• Waveform capturing and review waveforms<br>• Delivery of equipment and study files<br>• High-risk clinic support
Other observations and unforeseen circumstances:	• Delay in approval from provincial research committee resulting in a recruitment delay of 3 weeks<br>• Study team able to recruit at 2 clinics on the same day based on patient numbers<br>• Labour ward and ANC clinic team reported more foetal distress in screened high-risk women
**Site I**	
Stakeholder engagement:	• Hospital CEO, HOD, clinics, clinicians<br>• Meeting with hospital CEO, HOD and management<br>• Meeting with clinics and clinicians discussing BANC Plus and Umbiflow™<br>• Meeting with clinicians and HOD – discussions on Umbiflow™ and BANC Plus<br>• HOD actively involved with identifying study nurse and data clerk to join the team
Setting up the facilities:	• Both clinics had space available for a dedicated Umbiflow™ room
Composition of onsite study team:	• Study nurse, data clerk, hospital doctors<br>• Hospital team: HOD, Specialist, registrars
Training of study nurse and data clerk:	• Training in Pretoria (10 days)<br>• Practical at Study site A<br>• Electronic birth register data entry
Clinic arrangements for Umbiflow™ screening:	• Both clinic had space and multiple consultation rooms<br>• Umbiflow™ had a dedicated consultation room
Recruitment schedule:	• Monday and Thursday: Clinic 1 (CHC)<br>• Tuesday and Friday: Clinic 2
Day for high-risk clinic:	• Wednesday, run by Head of Department, specialists and registrars<br>• Dedicated abnormal RI clinic
Issues raised during virtual support:	• Digital Umbiflow™ interface:<br>- Testing software settings<br>- Adjusting the probe sound<br>• Stationary and equipment needs (printing paper, printer cable, study booklets, referral forms, and delivery forms)<br>• Recruiting numbers, data entry and referral pattern

Characteristics and processes	Site-specific description
Issues raised during onsite visits:	• Study files data entry and Doppler waveform review<br>• Progress with implementation of electronic birth register, and data entry<br>• Delivery of equipment and study files<br>• Taking completed files back to UP/SAMRC Unit for collation and RedCap entry<br>• High-risk clinic support
Other observations and unforeseen circumstances:	• Clinics have specialist visits weekly, this assisted with high-risk clinic booking.<br>• Local service-delivery protests in the area<br>• Provincial, district and DCST not involved in team work

TABLE S2. Umbi9 recruiting primary healthcare clinic characteristics

Indicator: clinics	Umbi9 implementation sites								
	A	B	C	D	E	F	G	H	I
**Clinic staff**									
Usual staff and nursing capacity available	✓	✓	✓	✓	✓	✓	✓	✓	✓
Necessary ANC training and skills	✓	✓	✓	✓	✓	✓	✓	✓	✓
**Recruitment facilities (clinics)**									
Accessible for Umbi-staff and patients	✓	✓	✓	✓	✓	✓	✓	✓	✓
Transport for Umbi-staff	✓	✓	✓	✓	✓	✓	✓	✓	✓
Feasible distance to hospital	✓	✓	✓	✓	✓	✓	✓	✓	✓
Separate dedicated CWDU screening area in the clinic	✓	**Integr**	**ANC2**	**ANC2**	✓	**Integr**	**ANC2**	✓	✓
**Umbi9 staff and equipment**									
Umbi-sister trained and available	✓	✓	✓	✓	✓	✓	✓*	✓	✓
Data capturer/clerk available	✓	✓	✓	✓	✓	✓	✓	✓	✓
Umbiflow™ probe	✓	✓	✓	✓	✓	✓	✓	✓	✓
Laptop	✓	✓	✓	✓	✓	✓	✓	✓	✓
Thermal printer	✓	✓	✓	✓	✓	✓	✓	✓	✓
**Referral route and services**									
Referral hospital	✓	✓	✓	✓	✓	✓	✓	✓	✓
Distance from hospital (accessibility)	**N**	**N&F**	**N**	**I**	**N&F**	**F**	**N**	**N&F**	**F**
EMS functional	✓	✓	✓	✓	✓	✓	✓	✓	✓
Patient transport from clinic to hospital	✘	✘	✘	✘	✘	✓	✘	✘	✘
Local transport (taxi, bus) available	✓	✓	✓	✓	✓	✓	✓	✓	✓

Indicator: clinics	Umbi9 implementation sites								
	A	B	C	D	E	F	G	H	I
**Data**									
Collection and recording	✓	✓	✓	✓	✓	✓	✓	✓	✓
Reporting of outcomes	✓	✓	✓	✓	✓	✓	✓	✓	✓
Data storage	✓	✓	✓	✓	✓	✓	✓	✓	✓
Regular audits	✓	✓	✓	✓	✓	✓	✓	✓	✓
PPIP use	✓	✓	✓	✓	✓	✓	✓	✓	✓
Maternity register	✓	✓	✓	✓	✓	✓	✓	✓	✓

* Original Umbi-sister resigned and additional Umbi-training was done with the new appointment

Integr = integrated with ANC consult done by Umbi-sister

ANCx2 = in the same area as ANC, but with Umbi9 sister doing the CWDU screening

N = near

F = far

I = intermediate (not too near and not too far)

Umbi, Umbiflow™
EMS, emergency medical services
PPIP, Perinatal Problem Identification Programme

TABLE S3. Umbi9 referral hospitals: inclusion criteria fulfilled

Indicator: hospitals	Umbi9 implementation sites								
	A	B	C	D	E	F	G	H	I
**Healthcare workers**									
Staff capacity in sufficient numbers	✓	✓	✓	✓	✓	✓	✓	✓	✓
Obstetrics doctor	✓	✓	✓	✓	✓	✓	✓	✓	✓
Regular sonographer as part of team	✓	✗	✗	✗	✗	✓	✗	✗	✗
Umbi-sister	✓	✓	✓	✓	✓	✓	✓	✓	✓
Data capturer/clerk	✓	✓	✓	✓	✓	✓	✓	✓	✓
Neonatal doctor	✓	✓	✓	✓	✓	✓	✓	✓	✓
**Services available**									
Maternity facility	✓	✓	✓	✓	✓	✓	✓	✓	✓
ANC high-risk clinic	✓	✓	✓	✓	✓	✓	✓	✓	✓
Neonatal facility	✓	✓	✓	✓	✓	✓	✓	✓	✓
**Site facility**									
Umbi-specific area	✓	✓	✓	✓	✓	✓	✓	✓	✓
Accessibility for patients	✓	✓	✓	✓	✓	✓	✓	✓	✓
Local transport for patients (taxi, bus)	✓	✓	✓	✓	✓	✓	✓	✓	✓
Referral system	✓	✓	✓	✓	✓	✓	✓	✓	✓
EMS availability	✓	✓	✓	✓	✓	✓	✓	✓	✓
Level of hospital	**R**	**R**	**R**	**R**	**R**	**R**	**PT**	**R**	**R**
**Equipment**									
Ultrasound with Doppler function	✓	✓	✓	✓	✓	✓	✓	✓	✓
Theatre available	✓	✓	✓	✓	✓	✓	✓	✓	✓
Blood products available	✓	✓	✓	✓	✓	✓	✓	✓	✓
ANC high-risk clinic	✓	✓	✓	✓	✓	✓	✓	✓	✓
Steroids available	✓	✓	✓	✓	✓	✓	✓	✓	✓
**Management protocols**									
Protocols and guidelines	✓	✓	✓	✓	✓	✓	✓	✓	✓
ESMOE drills	✓	✓	✓	✓	✓	✓	✓	✓	✓

**Indicator: hospitals**	**Umbi9 implementation sites**								
	**A**	**B**	**C**	**D**	**E**	**F**	**G**	**H**	**I**
**Neonatal unit**									
Registered professional nursing staff	✓	✓	✓	✓	✓	✓	✓	✓	✓
NICU	✓	✓	✓	✓	✓	✓	✓	✓	✓
Neonatal observation/nursery	✓	✓	✓	✓	✓	✓	✓	✓	✓
Equipment: care for >1kg neonate	✓	✓	✓	✓	✓	✓	✓	✓	✓
**Data**									
Collection and recording	✓	✓	✓	✓	✓	✓	✓	✓	✓
Reporting on outcomes	✓	✓	✓	✓	✓	✓	✓	✓	✓
Data storage and filling	✓	✓	✓	✓	✓	✓	✓	✓	✓
Regular audits	✓	✓	✓	✓	✓	✓	✓	✓	✓
PPIP	✓	✓	✓	✓	✓	✓	✓	✓	✓
Maternity birth register	✓	✓	✓	✓	✓	✓	✓	✓	✓

R = regional (hospital) PT = provincial tertiary (hospital)

Umbi, Umbiflow™
EMS, emergency medical services
ANC, antenatal care;
ESMOE, Essential Steps in Managing Obstetric Emergencies
NICU, neonatal intensive care unit
PPIP, Perinatal Problem Identification Programme

TABLE S4. Overview of activities of the central research team and the local study teams across the nine sites

Year: / Month: / Activity	2017												2018												2019											
	1	2	3	4	5	6	7	8	9	10	11	12	1	2	3	4	5	6	7	8	9	10	11	12	1	2	3	4	5	6	7	8	9	10	11	12
Site engagement																																				
Baseline assessment																																				
Site feasibility																																				
Umbiflow™ training																																				
EBR training																																				
BANC Plus																																				
Umbiflow screening																																				
Data collection																																				
Site visits and follow-up																																				

EBR, electronic birth register; BANC, basic antenatal care
